# Performance metrics of *in vitro* tests

**DOI:** 10.17179/excli2019-1693

**Published:** 2019-08-07

**Authors:** Florian Seidel

**Affiliations:** 1 Leibniz Research Centre for Working Environment and Human Factors, Ardeystr. 67, 44139-Dortmund, Germany

## ⁯

***Dear Editor,***

A frequent scenario concerning predictive *in vitro* tests in toxicology is that a compound is either tested as toxic or non-toxic *in vitro* and this prediction is then compared to the human *in vivo* situation for validation. The performance of such binary classification tests is assessed by established metrics, for example, sensitivity as the proportion of actual toxic compounds that were predicted as such; or sensitivity that measures the proportion of compounds that are non-toxic and were correctly predicted as non-toxic by the *in vitro* test. 

However, measures of the performance of binary classifications become suboptimal, when concentration or dose-dependent analyses are performed and the tests aim at predicting doses that cause an increased risk of toxicity *in vivo*. Recently, Albrecht et al. (2019[1]) addressed this challenge and established the Toxicity Separation Index (TSI) and Toxicity Estimation Index (TEI) as new performance metrics. Both, TSI and TEI, are calculated based on the projection of positive and negative test compounds onto a two-dimensional coordinate system. Here, the y-axis indicates the *in vivo* blood concentration - for example C_max_ - that results from a dosing schedule of a test compound, usually from therapeutic doses or from accidental overdoses. The x-axis represents the lowest concentration that causes a positive *in vitro* test result, also called *in vitro* alert. If the test differentiates well between toxic and non-toxic compounds, the toxic compounds will appear on top of the non-toxic substances in this presentation. The TSI is a continuous number that informs how well the test system differentiates between toxic and non-toxic compounds; a TSI of 1.0 indicates perfect separation, while a TSI of 0.5 represents a random result. The second recently introduced performance measure, the Toxicity Estimation Index (TEI), informs how well toxic blood concentrations *in vivo* can be estimated by the *in vitro* test system. The advantage of these new performance measures is that they can be used to optimize test systems. For example, the authors showed that the use of an EC_10_ instead of EC_50_ for cytotoxicity analysis in hepatocytes leads to a higher TSI. Moreover, TEI was improved, when gene expression was included into the test battery, meaning that the lower alert concentration of both, cytotoxicity and gene expression resulted in a better TEI than using the alert concentration of each test individually. Therefore, the TSI and TEI concept allows to modify a test and learn whether the modified version performs better than the original one. Of course, conclusions drawn from a training set of compounds need to be validated in an independent compound set to avoid overfitting. 

Currently, numerous activities are ongoing to predict *in vivo* toxicity by *in vitro* tests (Leist et al., 2017[10]; Vinken and Hengstler, 2018[18]), particularly in the fields of hepatotoxicity (Godoy et al., 2013[5], 2016[6]; Hammad, 2013[7]; Frey et al., 2014[4]; Jansen et al., 2017[8]), cardiotoxicity (Sampaio et al., 2016[14]; Chaudhari et al., 2016[2][3]), developmental toxicity (Rempel et al., 2015[13]; Krug et al., 2013[9]) and neurotoxicity (Sisnaiske et al., 2014[17]; Micheli et al., 2018[12]; Meléndez et al., 2019[11]; Shinde et al., 2015[15], 2016[16]). The novel performance metrics introduced by Albrecht et al. will help to objectify how well *in vitro* tests predict specific forms of toxicity *in vivo*. 

## Conflict of interest

The author declares no conflict of interest.

## References

[1] Albrecht W, Kappenberg F, Brecklinghaus T, Stoeber R, Marchan R, Zhang M (2019). Prediction of human drug-induced liver injury (DILI) in relation to oral doses and blood concentrations. Arch Toxicol.

[2] Chaudhari U, Nemade H, Gaspar JA, Hescheler J, Hengstler JG, Sachinidis A (2016). MicroRNAs as early toxicity signatures of doxorubicin in human-induced pluripotent stem cell-derived cardiomyocytes. Arch Toxicol.

[3] Chaudhari U, Nemade H, Wagh V, Gaspar JA, Ellis JK, Srinivasan SP (2016). Identification of genomic biomarkers for anthracycline-induced cardiotoxicity in human iPSC-derived cardiomyocytes: an in vitro repeated exposure toxicity approach for safety assessment. Arch Toxicol.

[4] Frey O, Misun PM, Fluri DA, Hengstler JG, Hierlemann A (2014). Reconfigurable microfluidic hanging drop network for multi-tissue interaction and analysis. Nat Commun.

[5] Godoy P, Hewitt NJ, Albrecht U, Andersen ME, Ansari N, Bhattacharya S, Bode JG (2013). Recent advances in 2D and 3D in vitro systems using primary hepatocytes, alternative hepatocyte sources and non-parenchymal liver cells and their use in investigating mechanisms of hepatotoxicity, cell signaling and ADME. Arch Toxicol.

[6] Godoy P, Schmidt-Heck W, Natarajan K, Lucendo-Villarin B, Szkolnicka D, Asplund A (2015). Gene networks and transcription factor motifs defining the differentiation of stem cells into hepatocyte-like cells. J Hepatol. J Hepatol.

[7] Hammad S (2013). Advances in 2D and 3D in vitro systems for hepatotoxicity testing. EXCLI J.

[8] Jansen PL, Ghallab A, Vartak N, Reif R, Schaap FG, Hampe J (2017). The ascending pathophysiology of cholestatic liver disease. Hepatology.

[9] Krug AK, Kolde R, Gaspar JA, Rempel E, Balmer NV, Meganathan K (2013). Human embryonic stem cell-derived test systems for developmental neurotoxicity: a transcriptomics approach. Arch Toxicol.

[10] Leist M, Ghallab A, Graepel R, Marchan R, Hassan R, Bennekou SH (2017). Adverse outcome pathways: opportunities, limitations and open questions. Arch Toxicol.

[11] Meléndez C, Muñoz P, Segura-Aguilar J (2019). DT-diaphorase prevents aminochrome-induced lysosome dysfunction in SH-SY5Y cells. Neurotox Res.

[12] Micheli L, Mattoli L, Maidecchi A, Pacini A, Ghelardini C, Di Cesare Mannelli L (2018). Effect of Vitis vinifera hydroalcoholic extract against oxaliplatin neurotoxicity: in vitro and in vivo evidence. Sci Rep.

[13] Rempel E, Hoelting L, Waldmann T, Balmer NV, Schildknecht S, Grinberg M (2015). A transcriptome-based classifier to identify developmental toxicants by stem cell testing: design, validation and optimization for histone deacetylase inhibitors. Arch Toxicol.

[14] Sampaio SF, Branco AF, Wojtala A, Vega-Naredo I, Wieckowski MR, Oliveira PJ (2016). p66Shc signaling is involved in stress responses elicited by anthracycline treatment of rat cardiomyoblasts. Arch Toxicol.

[15] Shinde V, Klima S, Sureshkumar PS, Meganathan K, Jagtap S, Rempel E (2015). Human pluripotent stem cell based developmental toxicity assays for chemical safety screening and systems biology data generation. J Vis Exp.

[16] Shinde V, Sureshkumar P, Sotiriadou I, Hescheler J, Sachinidis A (2016). Human embryonic and induced pluripotent stem cell based toxicity testing models: future applications in new drug discovery. Curr Med Chem.

[17] Sisnaiske J, Hausherr V, Krug AK, Zimmer B, Hengstler JG, Leist M (2014). Acrylamide alters neurotransmitter induced calcium responses in murine ESC-derived and primary neurons. Neurotoxicology.

[18] Vinken M, Hengstler JG (2018). Characterization of hepatocyte-based in vitro systems for reliable toxicity testing. Arch Toxicol.

